# Overexpression of the major facilitator superfamily efflux pump gene *nfa56470* mediates ciprofloxacin resistance in *Nocardia farcinica*

**DOI:** 10.3389/fcimb.2025.1708290

**Published:** 2025-12-10

**Authors:** Chenchen Si, Qiang Jiang, Poshi Xu, Tao Li, Shixuan Hua, Zhenjun Li

**Affiliations:** 1 Department of Medical Laboratory, Fuwai Central China Cardiovascular Hospital, Central China Fuwai Hospital of Zhengzhou University, Zhengzhou, Henan, China; 2 Department of Oncology, Henan Provincial People's Hospital, Zhengzhou University People's Hospital, Zhengzhou, Henan, China; 3 National Key Laboratory of Intelligent Tracking and Forecasting for Infectious Diseases, National Institute for Communicable Disease Control and Prevention, Chinese Center for Disease Control and Prevention, Beijing, China

**Keywords:** *Nocardia farcinica*, resistance mechanisms, ciprofloxacin, efflux pump, efflux pump inhibitors, major facilitator superfamily, porin gene

## Abstract

**Objectives:**

To explore the molecular basis of ciprofloxacin resistance in *N. farcinica*, providing a scientific basis for clinical antibiotic use and controlling the spread of resistant strains.

**Methods:**

We analyzed 20 *N. farcinica* strains. The quinolone resistance-determining regions (QRDRs) of *gyrA* and *gyrB* were sequenced and compared to the reference strain IFM 10152. The impact of efflux pumps was assessed by measuring changes in ciprofloxacin minimum inhibitory concentration (MIC) in the presence of three efflux pump inhibitors (EPIs). RT-qPCR was used to quantify the expression of 2 porin genes and 26 putative major facilitator superfamily (MFS) efflux pump genes, with and without ciprofloxacin induction.

**Results:**

Sequencing revealed no resistance-associated mutations in *gyrA* or *gyrB*. The ciprofloxacin MICs were significantly reduced (4- to 64-fold) upon exposure to EPIs, confirming efflux pump activity. Porin gene expression was modestly downregulation but did not correlate with resistance. Notably, three MFS efflux pump genes (*nfa56470*, *nfa29840*, and *nfa34160*) were significantly upregulated under ciprofloxacin pressure. Among these, *nfa56470* emerged as the most consistently and highly overexpressed gene in resistant strains.

**Conclusion:**

This study identifies the overexpression of specific MFS efflux pump genes, particularly *nfa56470*, as a primary mechanism of ciprofloxacin resistance in *N. farcinica*, supported by both gene expression data and functional EPI assays. This finding provides a clear target for future research into combating nocardial resistance.

## 1 Introduction

The genus *Nocardia* are filamentous, Gram-positive, aerobic actinomycete, weakly acid-fast bacteria, they are widely distributed in natural environments such as soil, humus, and water bodies. With the advancement of molecular identification techniques, the classification of *Nocardia* species has undergone continuous refinement. Currently, 251 species of *Nocardia* are now known to include 54 species associated with human infections (Parte et al., 2020). As opportunistic pathogens, they are capable of causing severe infections (Duggal Shalini and Chugh Tulsi, 2020), particularly pulmonary, cutaneous, and disseminated nocardiosis in both immunocompromised (Palomba et al., 2022) and immunocompetent individuals (Lv et al., 2023), which is associated with a high mortality rate averaging approximately 36.8% (Soueges et al., 2022).

Among the clinically relevant species, *N. farcinica* is frequently isolated and is recognized for its high virulence and pronounced multidrug-resistant phenotype (Qiu et al., 2022). This pathogen exhibits a high propensity for disseminated disease, typically initiating from pulmonary or cutaneous sites and spreading hematogenously to multiple organ systems including the central nervous system (Ravindra et al., 2021; Wang et al., 2024; Zhang et al., 2025). Recent reports, however, highlight an expanding clinical spectrum that includes intra-abdominal infections, such as liver and kidney abscesses, infectious diarrhea (Yang et al., 2024), and even bovine abortion (Kim et al., 2024). Its multidrug resistance and capacity for widespread dissemination solidify its status as a serious public health concern.

The wider adoption of advanced diagnostic tools, including matrix-assisted laser desorption/ionization time-of-flight mass spectrometry (MALDI-TOF MS) (Chudzik et al., 2024; Liu et al., 2024), metagenomic next-generation sequencing (mNGS) (Chen et al., 2025) and targeted next-generation sequencing (tNGS) (Lin SSW et al., 2024), has improved the detection and species-level identification of *Nocardia*, revealing a higher incidence than previously reported. Nevertheless, nocardiosis remains diagnostically challenging due to its nonspecific clinical presentation (Yetmar et al., 2025), which often resembles tuberculosis or fungal infections. This frequently leads to delayed diagnosis and inappropriate empirical antibiotic use, which in turn may exacerbate antimicrobial resistance.

Of particular concern is the rising resistance to ciprofloxacin among clinical isolates of *N. farcinica*. Surveillance studies have documented an increasing prevalence of ciprofloxacin resistant strains (Saullo and Miller, 2020; Wei et al., 2021; Wang et al., 2022; Singer et al., 2023), with some reports indicating susceptibility rates as low as 55% (Hershko et al., 2023). Despite this emerging trend, the molecular mechanisms underpinning ciprofloxacin resistance in *N. farcinica* remain incompletely characterized, limiting the development of targeted therapeutic strategies.

To address this critical knowledge gap, our study systematically investigates the genetic and phenotypic basis of ciprofloxacin resistance in *N. farcinica*. We focus on identifying key mutations in the QRDRs, evaluating the contribution of porin and efflux pump activity to the resistant phenotype. By elucidating these mechanisms, this work aims to provide a foundation for more effective management and treatment of *N. farcinica* infections.

## 2 Methods

### 2.1 Bacterial strains and MIC determination

A total of 20 *N. farcinica* strains was analyzed, including: 8 reference strains acquired from DSMZ (German Collection of Microorganisms and Cell Cultures) and 12 clinical isolates provided by the Chinese Center for Disease Control and Prevention (CCDC), all speciated through conventional methods combined with 16S rRNA gene sequencing.

Ciprofloxacin susceptibility was determined via Alamar Blue microdilution assays in 96-well plates. According to the Clinical and Laboratory Standards Institute (CLSI M24-A2) breakpoints, strains were defined as susceptible (MIC ≤1 μg/mL) or resistant (MIC ≥4 μg/mL). Based on these criteria, the strains were segregated into ciprofloxacin-resistant (CIP-R, n=10) and ciprofloxacin-sensitive (CIP-S, n=10) groups for subsequent analysis.

### 2.2 PCR amplification, DNA sequencing and mutation sites analyzing

Genomic DNA was extracted from all 20 *N. farcinica* strains using Bacterial Genomic DNA Extraction Kit (Tiangen Biotech, Beijing). Specific primers targeting QRDRs of gyrA and gyrB were designed with Primer Premier 5 software (detailed in **Table 1**), based on *N. farcinica* IFM 10152 retrieved from NCBI database. The polymerase chain reaction (PCR) amplification of QRDRs were carried out in a 30-μL reaction system. Purified PCR products were sequenced by the Sanger Biotech, and the resulting sequences were analyzed against corresponding sequences from NCBI GenBank using MEGA v6.06.

**Table 1:** Primers sequence and product sizes.

Primer	Annotation	Sequence (5’ to 3’)	Product sizes (bp)
*gyrA*	resistance gene	5’ GCATCGCCGCATCCTCTAC 3’ 5’ TGCCGTTGCTGCCGTTCA 3’	389
*gyrB*	resistance gene	5’ GCAACGAACACCTCACGCACTG 3’ 5’ CGCATGAACCGGAACAGC 3’	496
*nfa15890*	porin gene	5’ TGTGAAGTTCGTCCAGATCGA 3’ 5’ CGAGTAGTGGTCACCGATGA 3’	154
*nfa49590*	porin gene	5’ AGAGCGTGGTCTTCGAGTAG 3’ 5’ GGTGAAGTTCGTCCAGATCG 3’	167
*nfa11010*	MFS	5’ TCTTCACCGTCTGCTCGATC 3’ 5’ TGATCGAGGTGAGGAACAGG 3’	162
*nfa2050*	MFS	5’ GTTCATCGTCACGCTGTACC 3’ 5’ GGCAGGATCATCGTCAGGTA 3’	234
*nfa21830*	MFS	5’ CTACCTGCGGACTCGGTC 3’ 5’ GGTCTTCTTGCGGTGATG 3’	118
*nfa23460*	MFS	5’ GGATCGGACCGGGCAAGA 3’ 5’ ACAGCGCGGCGCACAACG 3’	114
*nfa23870*	MFS	5’ CGATCAACGCATCGACTA 3’ 5’ CAGCACGAACAGCACCAG 3’	160
*nfa25340*	MFS	5’ GCTCACCACCGACCTGGA 3’ 5’ CACCGAACCGACACCGAA 3’	166
*nfa25500*	MFS	5’ CCGATGGTGTTACCGGAGT 3’ 5’ CCTGGGCGATCTTCTTCAT 3’	118
*nfa29620*	MFS	5’ TGTTGCAGCCGTTGTACTTC 3’ 5’ CGGTATCCGGTCGATGAG 3’	131
*nfa29840*	MFS	5’ CGGCTACTACGACTGGGTCT 3’ 5’ ACGATCTTCAGCGGGATG 3’	123
*nfa29940*	MFS	5’ GTGGGGACTGATGGTGGT 3’ 5’ GGGTGTTGAAGCTGTTGG 3’	128
*nfa31040*	MFS	5’ CCTGCAGAACACCCAGCA 3’ 5’ CCAGCACCCGCCAAGAGC 3’	134
*nfa33330*	MFS	5’ ATGATCGTGAGCCGGATG 3’ 5’ AGAACTCCATCGTGGGACTG 3’	115
*nfa34160*	MFS	5’ ATCGCTCGGTGTGCTGATAC 3’ 5’ CGTCGGAATCGGTGAACA 3’	110
*nfa35800*	MFS	5’ GGTCGGTGTCGGTTTGAT 3’ 5’ AGGCGTGTTCGCGATGAT 3’	142
*nfa38830*	MFS	5’ GCACAAGCGCGGGGAGAC 3’ 5’ CGACGAACCACGGGAACA 3’	128
*nfa43970*	MFS	5’ AGCACCCTGCCGTTCATC 3’ 5’ CTCGTAGGCGGTTTCCGA 3’	119
*nfa44520*	MFS	5’ CAGGGCCTGGCAAAACCC 3’ 5’ CGATGAGCGGCACGAACG 3’	100
*nfa46310*	MFS	5’ CCTGCTGATGATGCTGTTC 3’ 5’ GCTGATGTATTCGGTGATGA 3’	130
*nfa47060*	MFS	5’ GATGACCCCGCAGACGAT 3’ 5’ CCACTCCCAGCTCAAGCC 3’	154
*nfa4770*	MFS	5’ GGCGGTGATGGCGATGTT 3’ 5’ GCGAAGCGGACGAAGCTG 3’	168
*nfa51130*	MFS	5’ GCCTCGTTGCCGTTGGAC 3’ 5’ GCGACACCCGGGTGGTGT 3’	130
*nfa56470*	MFS	5’ GTGCAACTCCTGCTCGTCA 3’ 5’ CGGTGAGCTTGATGGTCTG 3’	127
*nfa9390*	MFS	5’ AGTACCGCCTACACCCTG 3’ 5’ GATCCGAAGACGACCAAC 3’	110
*nfa21410*	MFS	5’ TGGTGCCCGAGCACGACC 3’ 5’ GCCAGCGCCACGAAGCGA 3’	120
*nfa33380*	MFS	5’ CGGGCACCGCGACCAATC 3’ 5’ GGCGCAACAACGTCAGCA 3’	120
*nfa33430*	MFS	5’ GCTCGTGTCGGTGATGCTCT 3’ 5’ CCGATGAACAGGCACAGGA 3’	132
*secA*	reference gene	5’ CCCAGTCCTCCACGTAGCCT 3’ 5’ CAGCAGCGCACCGTCATCT 3’	142

### 2.3 Impact of EPIs on ciprofloxacin susceptibility

The susceptibility of 20 *N. farcinica* strains to three EPIs: carbonyl cyanide m-chlorophenylhydrazone (CCCP), chlorpromazine (CPZ), and thioridazine (TZ) was assessed using the Alamar Blue microdilution assay in 96-well plates as before. The strains were then cultured with each EPI at subinhibitory concentrations, defined as 1/2 and 1/4 of its predetermined MIC of each EPI for each strain. After cultivation, ciprofloxacin MIC for each strain was redetermined.

### 2.4 Investigation on expression changes of porin genes and efflux pump genes

Based on the annotated whole-genome sequence of *N. farcinica* IFM 10152, we selected secA as reference gene, 2 porin genes and 26 putative drug efflux pump genes for analysis. Specific primers were designed with Primer Premier 5 software (detailed in **Table 1**).

Subsequently, the 20 *N. farcinica* strains were cultured in parallel under two conditions: without ciprofloxacin (control) and with subinhibitory ciprofloxacin (1/4 MIC). Total RNA was then extracted using the Qiagen Bacterial RNA Kit (Germany) and reverse-transcribed into cDNA. RT-qPCR was performed in 20 μL reaction mixtures assembled on ice, using cDNA as template. Amplification was performed on a 7500 FAST Real-Time PCR System using a two-step program (95°C denaturation; 60 °C annealing/extension). Gene expression levels were quantified via the 2^-ΔΔCT^ method, and a ≥4-fold change in expression (|ΔΔCT| ≥ 2) was considered significantly upregulated.

### 2.5 Statistical analysis

The reduction in MIC values of *Nocardia* strains after EPI application compared to baseline (without EPI) levels was analyzed by the Wilcoxon signed-rank test, with a P-value < 0.05 denoting statistical significance.

Differences in the relative expression of porin and efflux pump genes between the CIP-R and CIP-S groups were assessed using paired t-tests. Data were appropriately transformed and verified for normality (Shapiro-Wilk test) and homogeneity of variance (Levene’s test) prior to analysis, with a P-value < 0.05 denoting statistical significance.

## 3 Results

### 3.1 Mutation analysis of gyrA and gyrB in *N. farcinica*

**Figure 1:**
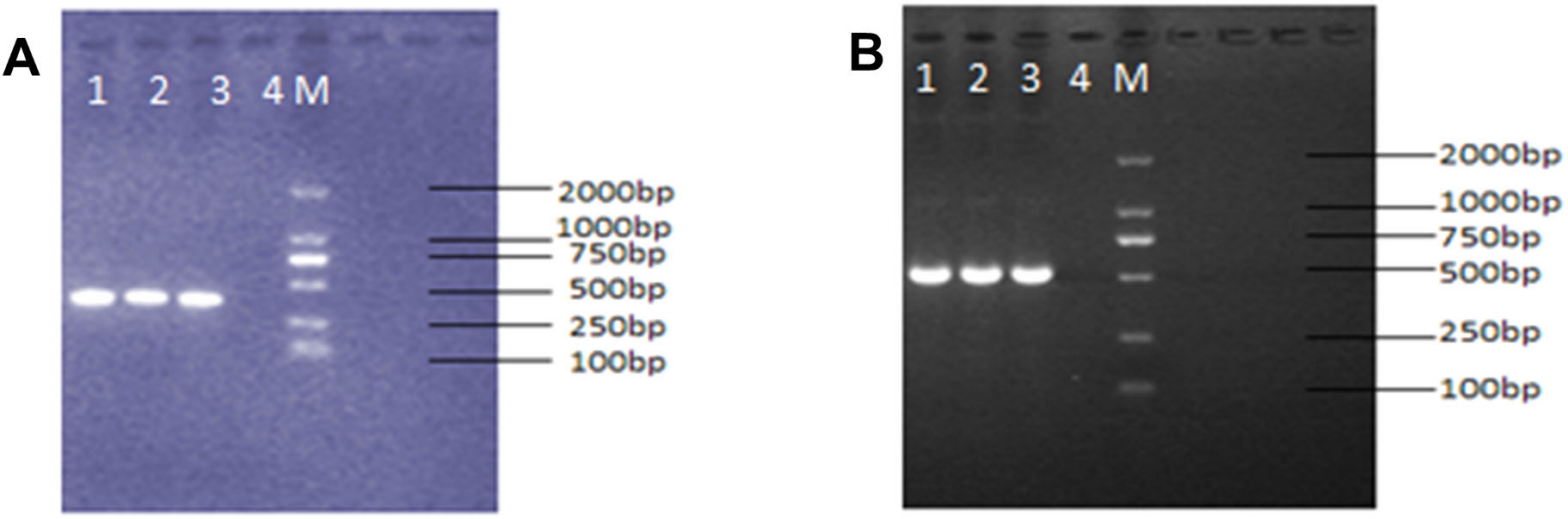
Electrophoresis diagram in *N. farcinica* strains. **(A)** gyrA, **(B)** gyrB; M: DL2000maker; 1,2,3: *N. farcinica*; 4: Negative control.

PCR amplification of gyrA and gyrB in all 20 *N. farcinica* strains yielded bands of ~400 bp and ~500 bp, respectively, matching predicted sizes (full electrophoresis presented in **Figure 1**).

Sequence alignment with *N. farcinica* IFM 10152 revealed a point mutation (C→G) at position 294 of gyrA in 6 strains (30%). This included two CIP-R (CDC25, CDC38) and four CIP-S strains (CDC27, CDC30, CDC31, CDC63). The mutation was synonymous, preserving arginine. Meanwhile, no mutations were detected in gyrB.

### 3.2 Analysis of the expression changes of porin genes

**Figure 2:**
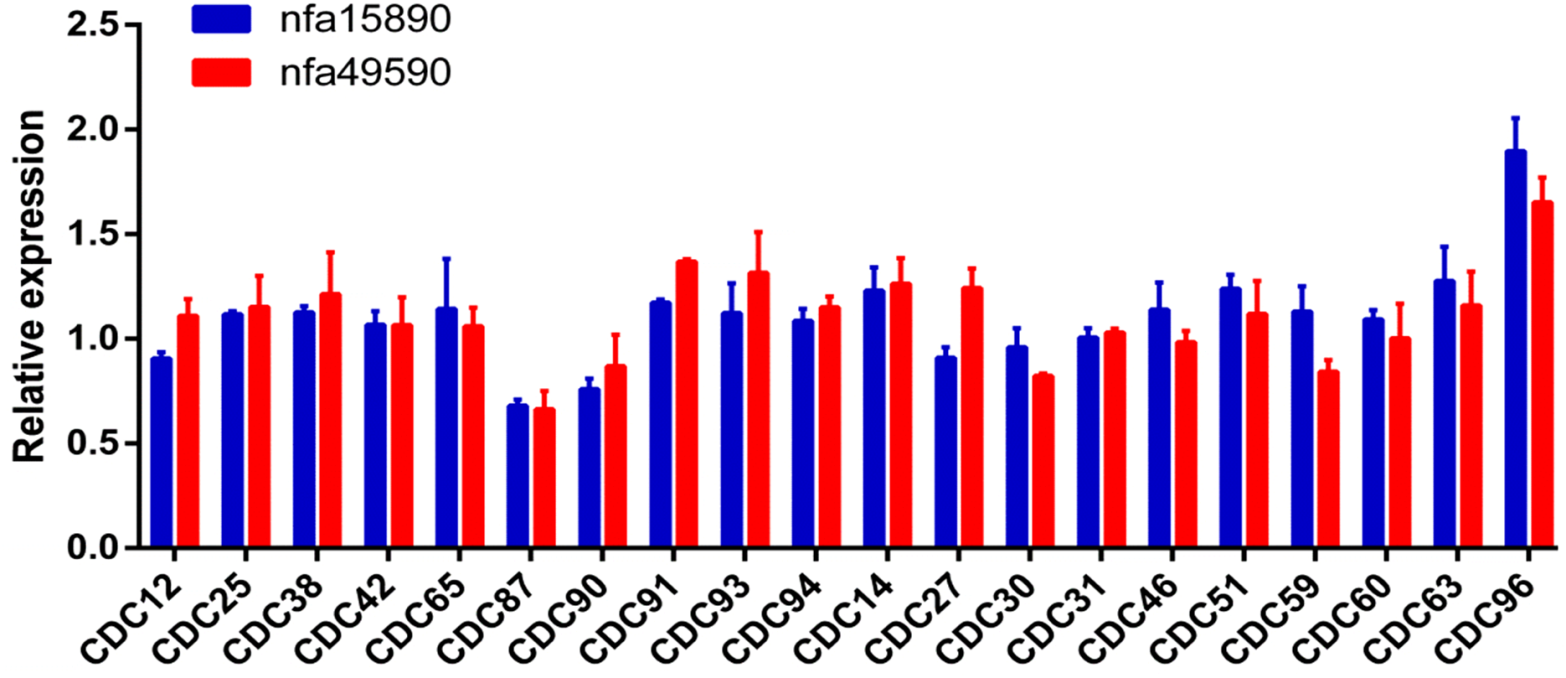
Downregulation in relative expression of porin genes. Horizontal Axis Label: *N. farcinica* Strains, Vertical Axis Label: Relative Expression.

**Figure 2** shows the downregulation of porin genes *nfa15890* and *nfa49590* across the tested strains. Among the 20 *N. farcinica* strains, CDC87 exhibited the mildest downregulation for both genes (~1.47- and ~1.52-fold), whereas CDC96 showed the largest (~1.89- and ~1.65-fold).

Compared to uninduced conditions, both porin genes were downregulated following exposure to subinhibitory ciprofloxacin (1/4 MIC), although the extent of downregulation was modest (<4-fold). The mean downregulation for *nfa15890* was 1.02-fold in the CIP-R group versus ~1.19-fold in the CIP-S group (P = 0.59). Similarly, *nfa49590* was downregulated by a mean of ~1.09-fold in CIP-R strains and ~1.11-fold in CIP-S strains (P = 0.68), with no statistically significant differences observed between the two groups.

### 3.3 Analysis of the expression changes of efflux pump genes

**Figure 3:**
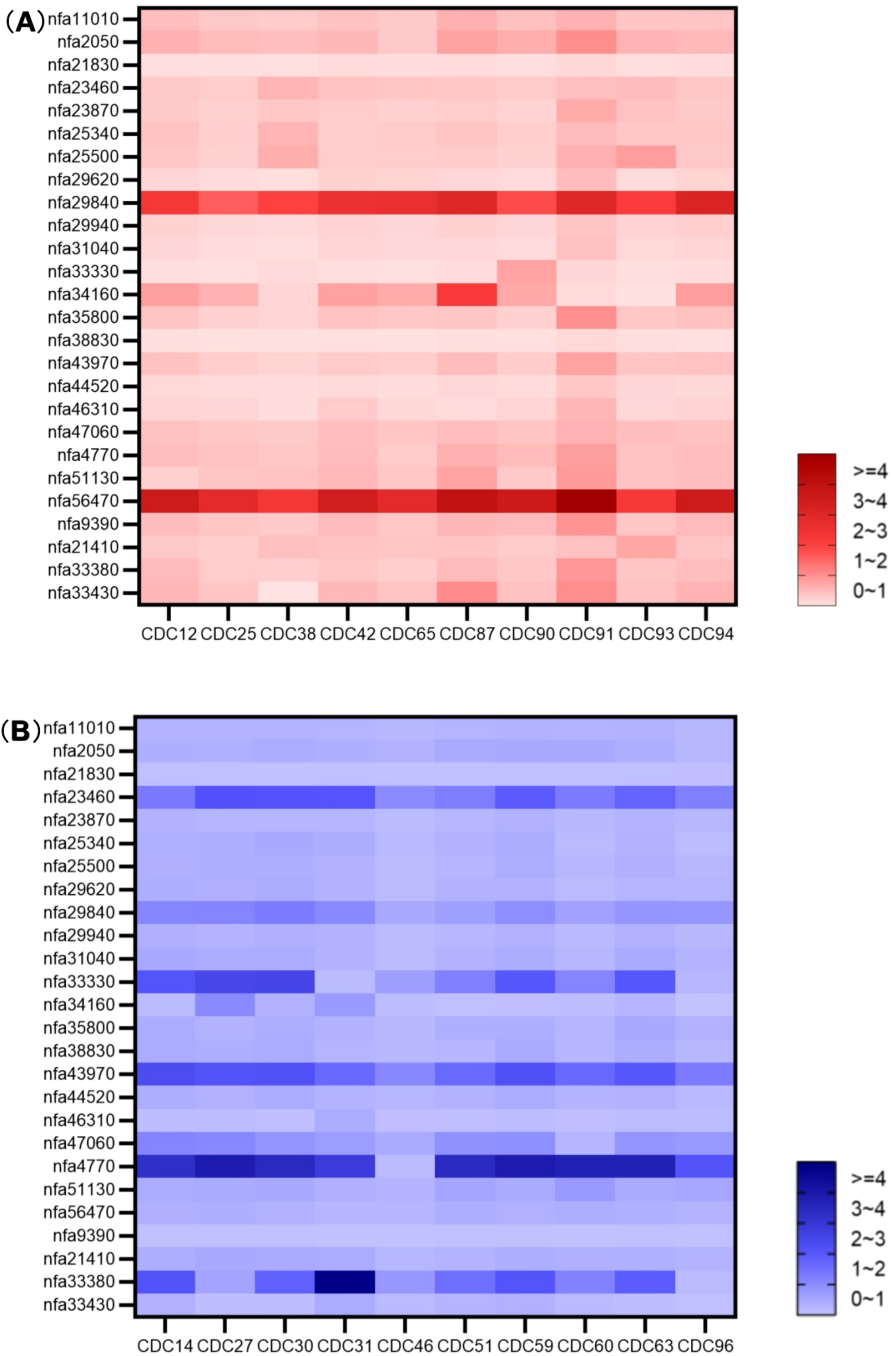
Heatmap of relative expression levels of efflux pump genes. **(A)** CIP-R group, **(B)** CIP-S group, Horizontal Axis Label: *N. farcinica* Strains, Vertical Axis Label: Efflux Pump Genes.

Expression of 26 MFS efflux pump genes in 20 *N. farcinica* strains under subinhibitory ciprofloxacin pressure (1/4 MIC) was analyzed relative to uninduced conditions, shown in the heatmap (**Figure 3**).

In CIP-R strains (n = 10), three efflux pump genes were significantly upregulated (≥4-fold): *nfa56470* (100% strains >4-fold), *nfa29840* (70% >4-fold; 30% 3-4-fold), *nfa34160* (10% >4-fold; 60% 1-2-fold; 30% 0-1-fold). As to CIP-S strains (n=10), five genes exhibited high expression: *nfa4770* (90% >4-fold; 10% 0-1-fold), *nfa43970* (80% >4-fold; 10% 3-4-fold; 10% 2-3-fold), *nfa23460* (50% >4-fold; 40% 3-4-fold; 10% 2-3-fold), *nfa33380* (50% >4-fold; 10% 3-4-fold; 20% 2-3-fold; 10% 1-2-fold; 10% 0-1-fold), *nfa33330* (50% >4-fold; 20% 2-3-fold; 10% 1-2-fold; 20% 0-1-fold). Most other genes showed less than 4-fold upregulation, with changes predominantly in the 0–2-fold range.

**Figure 4:**
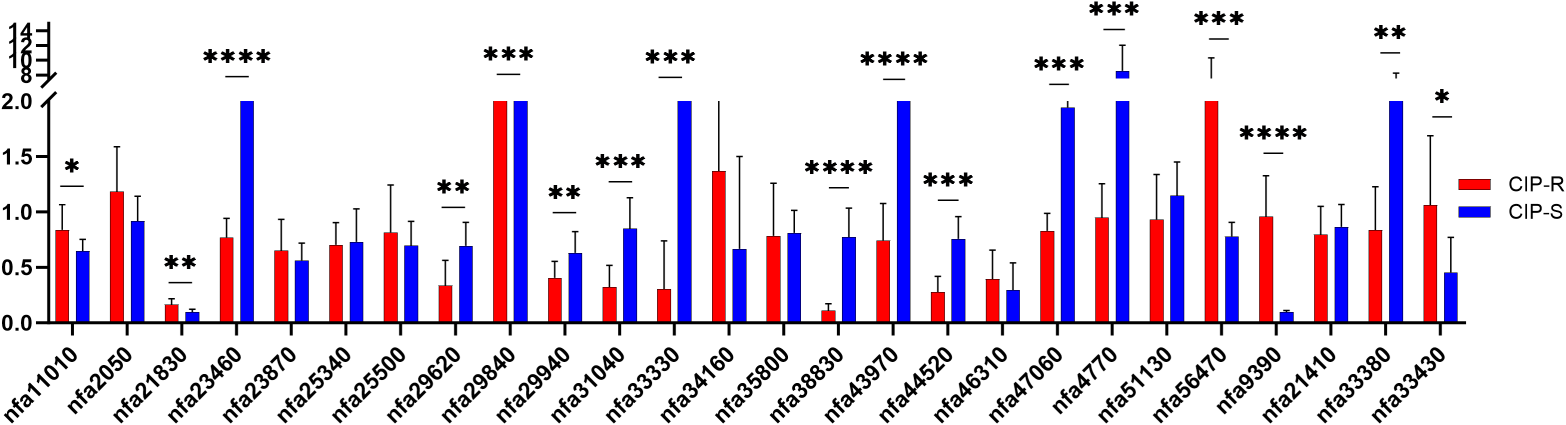
Comparison of the relative expression levels of efflux pump genes between the CIP-R and CIP-S groups. Horizontal Axis Label: Efflux Pump Genes, Vertical Axis Label: Relative Expression, Significance was defined as follows: *P < 0.05, **P < 0.01, ***P < 0.001, ****P < 0.000001.

Comparative statistical analysis between CIP-R and CIP-S groups revealed significant differential expression in 17 genes (**Figure 4**): *nfa21830*, *nfa23460*, *nfa29620*, *nfa29840*, *nfa29940*, *nfa31040*, *nfa33330*, *nfa38830*, *nfa43970*, *nfa44520*, *nfa47060*, *nfa4770*, *nfa56470*, *nfa9390*, *nfa33380*, *nfa11010* and *nfa33430*. Among these, *nfa23460*, *nfa38830*, *nfa43970*, and *nfa9390* exhibited highly significant differences (P < 0.000001). In contrast, genes such as *nfa2050*, *nfa23870*, *nfa25340*, *nfa25500*, *nfa34160*, *nfa35800*, *nfa46310*, *nfa51130*, and *nfa21410* showed no statistically significant differences (all P > 0.05).

## 4 Discussion

Ciprofloxacin, a second-generation fluoroquinolone antibiotic, is widely used in clinical practice to treat infections including those of skin and soft tissue wounds, urinary tract, respiratory tract, gastrointestinal tract as well as sexually transmitted diseases (STDs) (Gauba and Saxena, 2023; Rodrigues and Silva, 2025). Its broad-spectrum efficacy, high potency, low toxicity, cost-effectiveness, and lack of cross-resistance contribute to its effectiveness against pathogens especially multidrugresistant pathogens such as *Methicillin-resistant Staphylococcus aureus* (MRSA), *Klebsiella pneumoniae*, *Enterococcus* spp. (*E. faecium* and *E. faecalis*), *Campylobacter* spp., and *Acinetobacter baumannii (*Rodrigues and Silva, 2025; Saeful et al., 2025). Given the continuous increase in CIP-R *N. farcinica* in clinical settings, which once classified as susceptibility according to Category V, our study here aims to explore the molecular basis of ciprofloxacin resistance in *N. farcinica*, ultimately to provide scientific basis for the rational use of antibiotics and control the spreading of drug-resistant strains.

Quinolone antibiotics act bactericidally by inhibiting bacterial DNA replication, transcription, and topoisomerization, processes essential for survival. Chromosomally mediated resistance primarily occurs through mutations in drug targets: DNA gyrase (topoisomerase II) and topoisomerase IV (Gauba and Saxena, 2023; Rodrigues and Silva, 2025). DNA gyrase, a tetrameric enzyme composed of GyrA and GyrB subunits, regulates DNA supercoiling during replication and transcription. GyrA catalyzes DNA cleavage and religation, while GyrB provides energy via ATP hydrolysis (Mustaev et al., 2014). Topoisomerase IV (ParC_2_ParE_2_), homologous to DNA gyrase, resolves chromosomal entanglements during segregation. By binding to bacterial DNA and their target sites, quinolones induce conformational changes in DNA gyrase and/or topoisomerase IV, as well as in the broken DNA fragments. This blocks DNA relegation, induces double-strand breaks, halts replication, and causes cell death (Rodrigues and Silva, 2025).

The QRDR is a critical ~40-amino-acid domain within GyrA/ParC and GyrB/ParE for fluoroquinolone binding. Mutations in the QRDRs alter protein conformation and hydrophilicity, reducing drug affinity and preventing lethal complex formation while preserving enzyme function. Mutations within the QRDRs include single, double, and multiple gene mutations (Qian et al., 2020; Kato et al., 2024; Rezaei et al., 2024). Studies indicate that a single point mutation in the primary target reduces bacterial susceptibility to quinolones but does not confer full resistance; whereas multiple mutations in the primary target or concurrent mutations in secondary targets significantly enhance the level of resistance (Yu et al., 2020; Leyn et al., 2024).

Bioinformatic analysis of the *N. farcinica* IFM 10152 genome confirmed the presence of gyrA gene (locus 9691–12201, 2511 bp) and gyrB gene (locus 7529–9619, 2091 bp), yet no parC or parE homologs were detected. The QRDRs of gyrA and gyrB were mapped to amino acid residues 74–113 and 495–533, respectively. Primers targeting these regions were designed for PCR amplification, followed by amplicon purification and sequencing. A synonymous point mutation (C→G) was identified at nucleotide position 294 of gyrA, preserving the encoded arginine residue. This mutation occurred in both CIP-R (CDC25, CDC38) and CIP-S strains (CDC27, CDC30, CDC31, CDC63). No mutations were observed in the QRDR of gyrB. Therefore, mutations in the QRDRs of gyrA and gyrB are not associated with ciprofloxacin resistance in *N. farcinica*, consistent with Valdezate et al.’s findings (Valdezate et al., 2015).

Reduced membrane permeability and activation of efflux pumps constitute another major mechanism of bacterial antibiotic resistance (Sousa et al., 2022; Gauba and Saxena, 2023; Ghai, 2024; Saeful et al., 2025). Takumi Sato et al. investigated the relationship between the development of resistance and exposure duration to each fluoroquinolone by exposing *Escherichia coli* clinical isolates to fluoroquinolones *in vitro*. In strains exhibiting high fluoroquinolone resistance, acrA overexpression occurred alongside QRDR mutation (Sato et al., 2023). MacNair Craig R et al. reported that outer membrane perturbation reduces spontaneous rifampicin resistance and impairs biofilm formation (MacNair et al., 2020). With growing attention to antibiotic resistance, recent studies have revealed a strong link between resistance development and alterations in cell permeability. The outer membrane of Gram-negative bacteria restricts antibiotic entry, conferring intrinsic resistance to broad-spectrum antibiotics (Ghai and Ghai, 2018). Conversely, Gram-positive bacteria, lacking this outer membrane, typically remain susceptible. However, *Actinomycetales* (a Gram-positive order) exhibit intrinsic broad-spectrum resistance due to their mycomembrane (composed of long-chain mycolic acids and free lipids), which functionally resembles the Gram-negative outer membrane (Singh et al., 2015). Hydrophilic porin channels embedded within this mycomembrane facilitate diffusion of hydrophilic molecules (Sousa et al., 2022).

*N. farcinica* possesses an intrinsically dense membrane that restricts the permeability of nutrients as well as metabolic wastes and toxic compounds. This restricted permeability accounts for its characteristically slow growth rate and broad intrinsic resistance to antimicrobials. First identified in 1998, the outer membrane porin channel of *N. farcinica* is composed of two oligomeric subunits, NfpA and NfpB, and exhibits high sequence homology with the *Mycobacterium smegmatis* porin A (MspA) (Riess TL et al., 1998; Kläckta et al., 2011; Satheesan et al., 2024). Single-channel electrophysiology and liposome swelling assays demonstrate that this porin primarily facilitates diffusion of hydrophilic solutes, including sugars, amino acids, and select antibiotics (Ghai, 2024). In 2014, Pratik Raj Singh et al. demonstrated strong interactions between the *N. farcinica* porin channel and both cationic antibiotics (e.g. amikacin, kanamycin) and anionic antibiotics (e.g. ertapenem) (Singh et al., 2015).

Bioinformatic analysis of the *N. farcinica* IFM 10152 genome identified two porin genes: *nfa15890* (locus 1741453–1742127; 675 bp) and *nfa49590* (locus 5175668–5176342; 675 bp). Both genes exhibit high homology to the MspA family, as confirmed by protein homology prediction and automated computational analysis. Under subinhibitory ciprofloxacin exposure (1/4 MIC), the expression of both porin genes showed no significant downregulation (<2-fold change) and did not differ between CIP-R and CIP-S strains. These results indicate that altered membrane permeability contributes only marginally to ciprofloxacin resistance in *N. farcinica*.

The efflux pump, a critical component of the membrane transport system, mediates essential cellular functions beyond substrate extrusion. It facilitates nutrient uptake, expels metabolic wastes and xenobiotics, and contributes to intercellular communication through selective secretion of biomolecules (Henderson et al., 2021; Cruz et al., 2024). Collectively, these mechanisms enable cellular homeostasis amidst environmental fluctuations. Currently, six families of chromosomally encoded bacterial efflux pumps have been identified and relevant to antimicrobials. They are classified as multidrug and toxic compound extruder (MATE), major facilitator superfamily (MFS), small multidrug resistance (SMR), resistance nodulation division (RND), ATP-binding cassette (ABC) and proteobacterial antimicrobial compound efflux family (PACE) (Kabra et al., 2019; Garcia et al., 2022). In Gram-negative bacteria, RND pumps primarily mediate drug resistance, whereas MFS pumps dominate in Gram-positive species. Among them MFS is an ancient, large, and diverse group of membrane transporters found across bacteria, plants, and mammals (Pasqua et al., 2021). Its approximately 5,000 sequenced members span 58 distinct families and transport small molecules such as sugars, anions, drugs, metabolites, esters, oligosaccharides, and organophosphates (Pasqua et al., 2019). Structurally, MFS proteins consist of a single polypeptide chain (400–600 amino acids) with 12–14 transmembrane helices and cytoplasmic N- and C-terminal domains (Kumar et al., 2020).

MFS efflux pumps confer antibiotic resistance in several bacterial species. Leus et al. propose that MFS transporters AmfB and AmfD, along with periplasmic membrane fusion proteins AmfA and AmfC, enhance stress survival in *A. baumannii* by modulating cell envelope permeability (Leus et al., 2023). Li et al. characterize SA09310 as a tetracycline efflux pump conferring tetracycline resistance in *S. aureus (*Li et al., 2023). Separately, Li et al. report that *Mycobacterium tuberculosis* Rv0191 functions as a MFS efflux pump regulated by Rv1353c (Li et al., 2019), while Singh et al. identify Rv1634 as a multidrug transporter in the same superfamily (Singh and Akhter, 2021). Therefore, we selected 26 putative MFS genes for investigation through integrated analysis of NCBI and TransportDB databases. NCBI annotations confirmed these genes were identified via protein homology prediction and automated computational analysis. TransportDB further classified them as MFS transporters with predicted antibiotic substrate specificity.

To determine the contribution of efflux pumps to ciprofloxacin resistance, we used EPIs and observed a marked reduction (4- to 64-fold, P < 0.0001) in ciprofloxacin MIC across all tested strains, confirming that active efflux significantly modulates susceptibility. To pinpoint the specific genetic determinants, we analyzed the expression profiles of 26 putative MFS efflux pump genes. Under ciprofloxacin induction, three genes (*nfa56470*, *nfa29840*, and *nfa34160*) were significantly upregulated in the CIP-R group. We speculate that the distinct roles of these pumps underlie the resistant phenotype. Specifically, *nfa56470* appears to be overexpressed in all CIP-R strains, providing a baseline level of protection. Meanwhile, *nfa29840* and *nfa34160* might be more efficiently recruited or rapidly induced upon ciprofloxacin exposure, offering an inducible defense that synergizes with the constitutive mechanism. This combination could be crucial for achieving the high-level resistance observed.

A more complex picture emerged from the analysis of CIP-S strains, where genes like *nfa4770*, *nfa43970*, *nfa23460*, *nfa33380* and *nfa33330*, also exhibited high expression. This suggests a conserved, broad-spectrum stress response is activated upon antibiotic challenge. However, the fact that these strains remain susceptible implies that the mere activation of an efflux response is insufficient to confer clinical resistance. We hypothesize that this could be because these particular pumps have a low intrinsic affinity for ciprofloxacin, rendering them inefficient despite high expression. Alternatively, their expression level, even when induced, may not reach the threshold required for resistance without concomitant genetic adaptations, such as mutations in regulatory elements or synergistic changes in membrane permeability, that are present in the resistant strains.

A critical synthesis of the relative contributions of each investigated pathway reveals that efflux pump overexpression is the dominant mechanism of ciprofloxacin resistance in our strain set. This is strongly supported by the profound MIC reduction with EPIs and the marked upregulation of specific pump genes in resistant strains. The contribution of porin downregulation, while observed, is likely secondary. The modest (<4-fold) decrease in porin gene expression, which did not differ significantly between resistant and susceptible groups, may play a subtle synergistic role by reducing drug influx and thereby enhancing the efficiency of the overexpressed efflux pumps. In contrast, the role of target gene mutations appears negligible, as no resistance-conferring mutations were found in the QRDRs of gyrA or gyrB.

## 5 Conclusion

In conclusion, this study establishes that the overexpression of specific MFS efflux pumps represents the primary mechanism of ciprofloxacin resistance in *N. farcinica*, with *nfa56470* identified as the cornerstone alongside two additional key genes, *nfa29840* and *nfa34160*. The paradoxical efflux activation observed in susceptible strains underscores the complexity of bacterial stress responses, demonstrating that the identity and regulatory context of the pump, rather than its mere presence, ultimately determine the resistance outcome. While our findings provide crucial scientific evidence for guiding clinical antibiotic use and containing resistant strains, the protein structures, detailed functions, and precise mechanisms of action for these newly identified efflux pumps remain largely uncharacterized, warranting further investigation to fully elucidate this resistance pathway.

## Data Availability

The original contributions presented in the study are included in the article/**Supplementary Material**. Further inquiries can be directed to the corresponding author.
