# Supplementary material for: Overexpression of the major facilitator superfamily efflux pump gene nfa56470 mediates ciprofloxacin resistance in Nocardia farcinica
Source: Front Cell Infect Microbiol. 2025 Dec 10;15:1708290. doi: 10.3389/fcimb.2025.1708290 (PMC12727967; doi:10.3389/fcimb.2025.1708290)
Supplement: Supplementary file 1 [file Table1.docx]

***Supplementary materials***

1. **Supplementary Tables**

**MIC Determination**

MIC of ciprofloxacin and three EPIs: CCCP, CPZ, and TZ, against 20 *N. farcinica* strains are documented in Table 1.

| Strain | Source | phenotype | MIC Values（μg/ml） | | | |
| --- | --- | --- | --- | --- | --- | --- |
|  |  |  | CIP | CCCP | CPZ | TZ |
| CDC12 | DSMZ | R | 4 | 16 | 32 | 8 |
| CDC25 | Clinical | R | 4 | 16 | 16 | 8 |
| CDC38 | Clinical | R | 4 | 16 | 16 | 8 |
| CDC42 | Clinical | R | 4 | 8 | 16 | 8 |
| CDC65 | Clinical | R | 8 | 16 | 32 | 8 |
| CDC87 | DSMZ | R | 4 | 16 | 32 | 8 |
| CDC90 | DSMZ | R | 4 | 16 | 16 | 8 |
| CDC91 | DSMZ | R | 8 | 16 | 32 | 8 |
| CDC93 | DSMZ | R | 8 | 16 | 32 | 8 |
| CDC94 | DSMZ | R | 4 | 8 | 16 | 8 |
| CDC14 | DSMZ | S | 1 | 4 | 32 | 32 |
| CDC27 | Clinical | S | 0.5 | 4 | 32 | 16 |
| CDC30 | Clinical | S | 0.5 | 2 | 32 | 16 |
| CDC31 | Clinical | S | 0.5 | 2 | 32 | 16 |
| CDC46 | Clinical | S | 0.5 | 8 | 32 | 16 |
| CDC51 | Clinical | S | 0.5 | 4 | 32 | 16 |
| CDC59 | Clinical | S | 0.5 | 8 | 64 | 16 |
| CDC60 | Clinical | S | 1 | 4 | 32 | 16 |
| CDC63 | Clinical | S | 0.5 | 4 | 32 | 16 |
| CDC96 | DSMZ | S | 0.5 | 2 | 32 | 8 |

Table 1 MIC Values of CIP and Three EPIs

R: resistance, S: sensitivity, CIP: ciprofloxacin, CCCP: carbonyl cyanide m-chlorophenylhydrazone, CPZ: chlorpromazine, TZ: thioridazine.

1. **Supplementary Figures**

**Impaction of EPIs on Ciprofloxacin Susceptibility**

Pretreatment with subinhibitory concentrations (1/2 and 1/4 MIC) of each EPI significantly reduced the ciprofloxacin MIC against *N. farcinica* strains to 0.125 μg/mL (4- to 64-fold reduction; P < 0.0001, Wilcoxon signed-rank test). For example, in strain CDC12 (Figure 1), EPI pretreatment reduced the ciprofloxacin MIC from 4 μg/mL to 0.125 μg/mL (32-fold reduction; Figure 2). These results demonstrate that EPIs substantially increase *N. farcinica* susceptibility to ciprofloxacin.


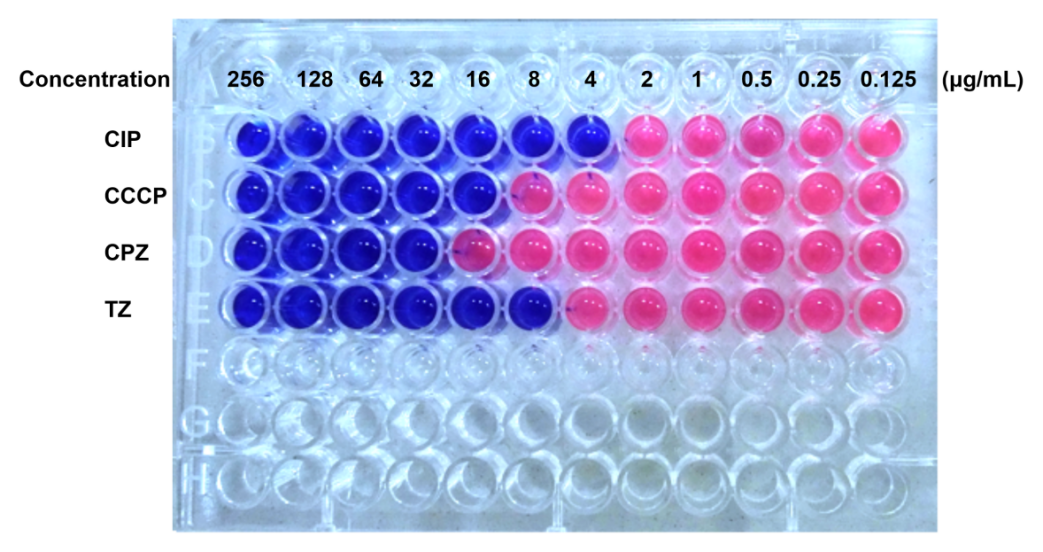


Figure 1 MIC Values of ciprofloxacin and three EPIs against *N. farcinica* CDC12


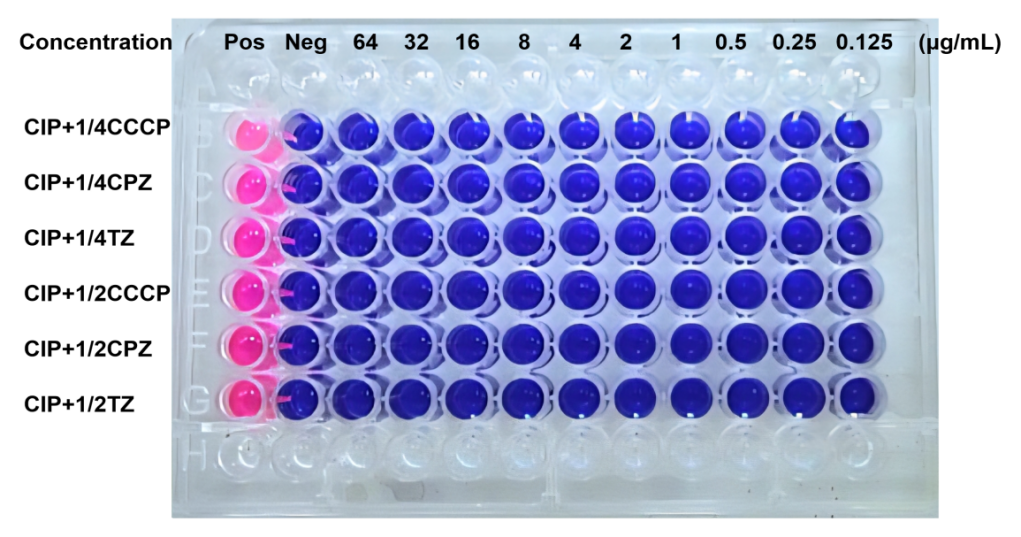


Figure 2 MIC Values of ciprofloxacin after cultured in the presence of each EPI in *N. farcinica* CDC12

**Sequence Analysis of gyrA and gyrB Genes in *Nocardia farcinica***

Sequence data of gyrA and gyrB were analysed by comparison with sequences obtained from NCBI GenBank using MEGA v6.06(Figure 3).


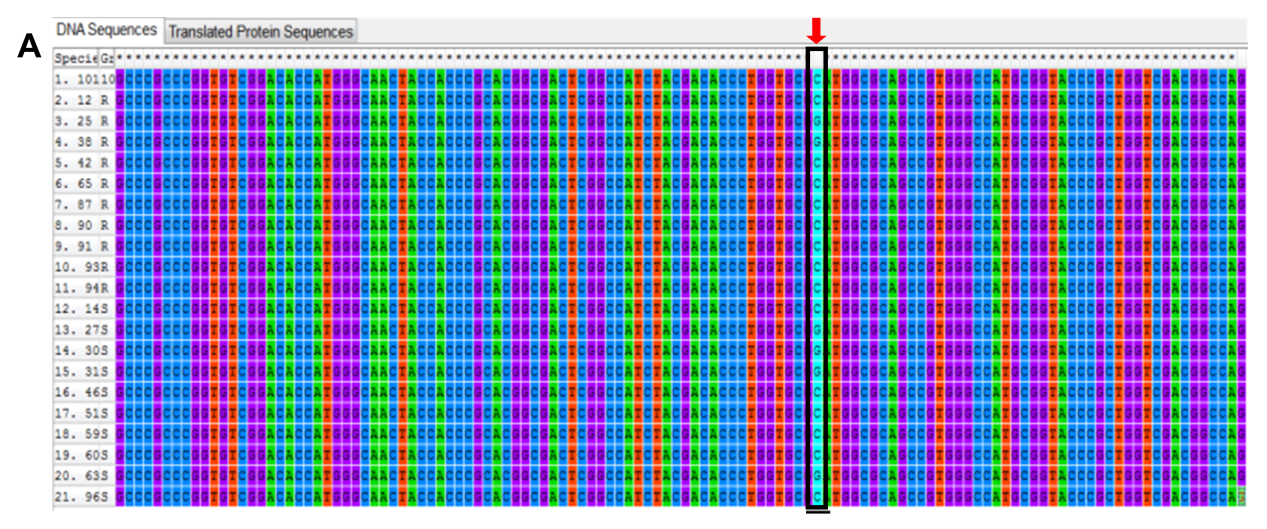

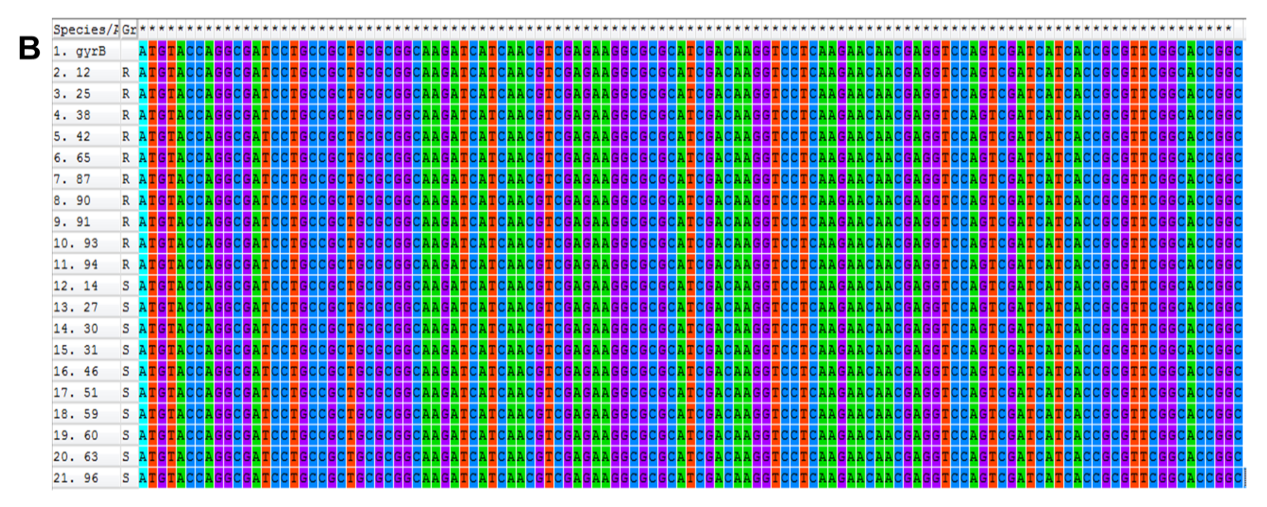


Figure 3 Alignment Results of Gene Sequences in *N. farcinica*

A: gyrA; B: gyrB
